# Helping the Blind to Get through COVID-19: Social Distancing Assistant Using Real-Time Semantic Segmentation on RGB-D Video

**DOI:** 10.3390/s20185202

**Published:** 2020-09-12

**Authors:** Manuel Martinez, Kailun Yang, Angela Constantinescu, Rainer Stiefelhagen

**Affiliations:** 1 Institute for Anthropomatics and Robotics, Karlsruhe Institute of Technology, 76131 Karlsruhe, Germany; manuel.martinez@kit.edu (M.M.); rainer.stiefelhagen@kit.edu (R.S.); 2 Study Centre for the Visually Impaired, Karlsruhe Institute of Technology, 76131 Karlsruhe, Germany; angela.constantinescu@kit.edu

**Keywords:** computer vision for the visually impaired, social distancing, semantic segmentation

## Abstract

The current COVID-19 pandemic is having a major impact on our daily lives. Social distancing is one of the measures that has been implemented with the aim of slowing the spread of the disease, but it is difficult for blind people to comply with this. In this paper, we present a system that helps blind people to maintain physical distance to other persons using a combination of RGB and depth cameras. We use a real-time semantic segmentation algorithm on the RGB camera to detect where persons are and use the depth camera to assess the distance to them; then, we provide audio feedback through bone-conducting headphones if a person is closer than 1.5 m. Our system warns the user only if persons are nearby but does not react to non-person objects such as walls, trees or doors; thus, it is not intrusive, and it is possible to use it in combination with other assistive devices. We have tested our prototype system on one blind and four blindfolded persons, and found that the system is precise, easy to use, and amounts to low cognitive load.

## 1. Introduction

**Figure 1:**
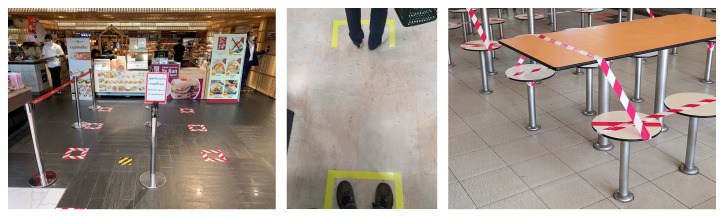
Visual markings placed to assist in the maintaining of physical distance. Source: Wikimedia commons. Markings come in very different shapes and cannot be perceived by blind or visually impaired people.

Several measures are currently in place to slow the spread of the COVID-19 pandemic. One of these measures, named social distancing or physical distancing, aims to prevent the transmission of the disease by keeping a minimum physical distance between people. The rules of social distancing vary between regions; for example, in Germany, it is required to maintain a distance of at least 1.5 m between people in shopping malls, and there are often visual cues placed on the floor to help people to assess the required distance and which act as a reminder, as seen in Figure 1.

However, blind and visually impaired people are sometimes unable to perceive the distance between themselves and nearby persons; furthermore, they are unable to see the visual cues. Additionally, due to the haste in implementing such measures, most social distancing markings were created without considering accessibility for blind and visually impaired persons.

This situation negatively impacts the ability of blind people to navigate pubic environments and interact with society. Failure to respect social distancing marks often creates conflicts. While in most situations sighted people can assist the visually impaired, in these situations, this is not always the case, and stressful situations occur on a regular basis.

We corresponded with our colleagues at the Study Centre for Visually Impaired Students (https://www.szs.kit.edu/english/index.php) at KIT. They rate social distancing as one of the major ways in which COVID-19 is impacting the visually impaired community, with many blind people opting to stay at home at all costs.

**Figure 2:**
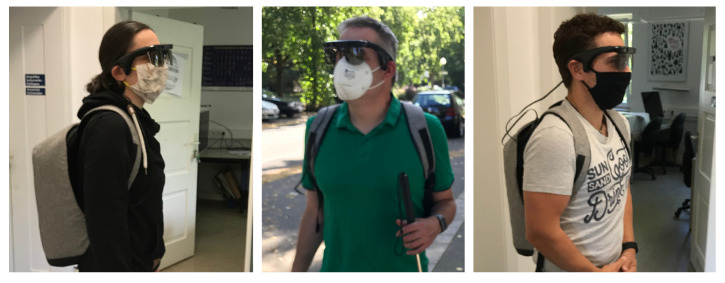
Incidences of participants using the system to maintain social distancing.

To help blind users to adapt to the current situation, we designed a system that makes them aware of nearby persons in front of them; see Figure 2. For the perception part of the system, we use KR-Vision [1] glasses. These integrate a depth camera, a color camera and bone-conducting headphones to provide audio feedback. The color camera is used to feed a deep learning-based real-time semantic segmentation algorithm [2] that is trained on the Mapillary Vistas dataset [3]. The output of the semantic segmentation algorithm provides a pixel-wise segmentation mask in which persons are detected. While there are efficient object detection methods [4,5] that can be used for person detection with bounding boxes, we consider that the pixel-wise segmentation mask helps to generate more accurate sound feedback. We map the depth camera to the output of the fast segmentation algorithm to detect the distance of the perceived persons. Finally, if persons are detected within the predefined selected range, which defaults to 50 cm to 150 cm, our system outputs a beeping sound to alert the user.

We use the Robotic Operating System (ROS) [6] to connect the different software components in our system. ROS allows the easy and robust interfacing of components written in different languages and frameworks. In this case, it allows us to interface the camera recording component, written in C++, with the deep learning component, which is a native PyTorch application written in Python, and back to the audio feedback module, also written in C++.

The user interface is designed to be as non-intrusive as possible. The bone-conducting headphones do not occlude the ears of the users, allowing them to continue to hear ambient sound. We use a sonification-based warning approach: if no person is detected in the immediate vicinity of the user, no sound is produced. This allows our system to be integrated on top of other assistive technologies without affecting their use. The sonification method used is parameter mapping. The sound output is modulated in volume and frequency: the confidence of the detection modulates the volume of the response, while the urgency is modulated in pitch. Thus, if a person is detected for a long time, the pitch of the response increases to raise the urgency level.

We tested the system and the interface on one blind and four blindfolded users and we found that it works well in both indoor and outdoor environments. Additionally, it was well received by the users, who found it intuitive to use. We measured the mental workload of the system using the Raw NASA-TLX, abbreviated as the Raw NASA Task Load Index (RTLX) [7], a simpler version of the initial NASA-TLX test [8]. The results show the results of the system with a low cognitive load. One major drawback that the sighted users (not the blind user) noted during the user study was the rather slow response time, which was solved in a further iteration of the software.

In this system, we leveraged technologies that have reached a high level of maturity only in recent years: using ROS to communicate between computing nodes, using RGB-D cameras to visually perceive the world around us and using Deep Learning to process images coming from an unstructured human environment. The maturity of these concepts has allowed us to design a successful prototype quickly after the problem was recognized and to focus on the system design, usability and user interaction.

## 2. Related Work

### 2.1. Hazard Avoidance for the Visually Impaired with RGB-D Sensors

Detecting hazards from wearable cameras requires the modeling of the spatial environment in front of the user. This has been done explicitly using stereo cameras [9] or implicitly using conditional random fields [10]. However, RGB-D cameras have become popular for this task due to their light weight, cost-effectiveness and the capacity to acquire 3D information in real time [11]. Aladren et al. [12] showed a system that combines the color and depth information of a RGB-D camera to provide long-range obstacle-free paths. Yang et al. [13] enhanced the depth maps from an RGB-D camera to expand the detection range of traversable areas and improve path planning. Wang et al. [14] detected known obstacles from a chest-worn stereo camera. To overcome the range limitations of RGB-D cameras, some authors have augmented them using additional sensors such as ultrasonic sensors and millimeter-wave radars [15,16]. Furthermore, water hazard detection [17] and small obstacle avoidance [18,19] have also been addressed.

Martinez et al. [20] proposed the leveraging of the recently developed technologies for autonomous vehicles to develop assistive tools for visually impaired people. Specifically, they performed a proof-of-concept study by using the *stixel* algorithm [21] to represent the obstacles in front of the users. Wang et al. [22] further combined the geometric layouts based on *stixel* representations and pixel-wise semantics predicted by a real-time segmentation network. They constructed an environment perception and navigation assistance system with a wearable RGB-D camera. Bai et al. [23] mounted a RGB-D sensor on a pair of eyeglasses and designed a lightweight convolutional neural network (CNN)-based 2.5D object recognition module for deployment on a smartphone, providing obstacle category, location and orientation information. Kajiwara and Kimura [24] designed an object identification and route recommendation system based on human flow for the visually impaired. Specifically, they used the OpenPose model [25] to detect human skeletons using a RGB-D camera, where the depth maps enabled the localization of the pedestrian’s skeleton trunks for human flow avoidance. Recently, Dimas et al. [26] also devised a pair of smart glasses based on an RGB-D sensor and performed the uncertainty-aware modeling of obstacle risk assessment for the visually challenged. While products such as the Bat Orientation Guide [27] allows people or moving objects to be followed at a constant distance, they cannot handle the social distancing problem in unstructured environments. In this work, with vision-based perception, we took steps beyond conventional obstacle avoidance technologies and explicitly aimed to assist the visually impaired to follow social distancing, which has not been addressed by any previous work in the literature.

### 2.2. Semantic Segmentation to Help the Visually Impaired

Semantic segmentation has emerged as a powerful technology to unify the perception tasks desired by navigation assistance applications. Thanks to the emergence of large datasets [3] and the architectural advances of deep models [28,29], modern networks are able to perform semantic segmentation both accurately and efficiently. While it has been widely used in autonomous driving systems [30], semantic segmentation has been less widely explored for helping blind people. In this line, Yang et al. [31,32] seized real-time semantic segmentation to provide traversability awareness and multi-class terrain sensing for visually impaired people, which are the most essential tasks for assisted navigation. Cao et al. [33] designed a lightweight semantic segmentation network to achieve the rapid detection of blind roads and sidewalks in a unified way, which was similarly achieved in [34] for intersection perception, covering the detection of crosswalks and pedestrian crossing lights. Mehta et al. [35] took advantage of the spatial and temporal attributes of the objects extracted from semantic segmentation maps to identify the most walkable direction in a scene. Watson et al. [36] the prediction of footprints from RGB images by including the detection of hidden walkable surfaces, thus surpassing semantic segmentation, which only handles visible traversable areas.

Lin et al. [37] developed a wearable assistive system by generating collision-free instructions with touchscreen interactions to fully make use of semantic segmentation maps. In [38,39], instance-specific semantic segmentation was leveraged to help blind people to recognize objects in their surroundings by using state-of-the-art instance segmentation models such as Mask R-CNN [40]. Mao et al. [41] employed a panoptic segmentation model, named Seamless Scene Segmentation [42], to unify the segmentation of objects that are of critical relevance to the perception required by visually impaired people. However, it takes more than one second to yield a complete segmentation for a single frame. In our work, instead of relying on such accurate yet computation-intensive models, we use a real-time semantic segmentation algorithm, DS-PASS [2], that is both efficient, robust and can be deployed in portable systems. In addition, many of the previous systems [33,35,36] did not exploit depth cues, making the extracted semantics less informative for navigation assistance. In this work, we perform fast pixel-wise semantic segmentation with associated dense depth information from a RGB-D sensor to help blind people to maintain safety-critical social distancing. To the best of our knowledge, the visual social distancing problem [43] has only been defined in monitoring applications. Our work differs fundamentally from the previous study, as we aim to provide blind people with a situational awareness of social distancing with an egocentric, wearable vision system.

## 3. System

### 3.1. Hardware Components

**Figure 3:**
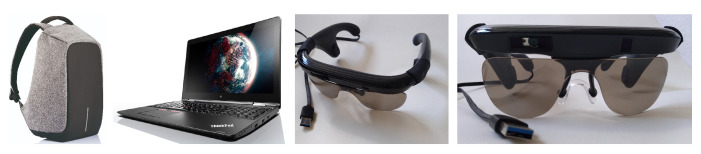
Hardware components used in our prototype. From left to right: a lightweight backpack, a Lenovo Thinkpad Yoga 14 laptop and the KR-Vision glasses. The glasses house bone-conducting headphones, better seen in the third image from the left as the protuberances in the temple pieces. The RGB-D camera, made by Intel, is housed inside the thick bezel above the lenses, best seen in the rightmost image.

Our prototype has very few hardware parts; see Figure 3. The perception component, based on the KR-Vision glasses (KR-Vision Technology, Hangzhou, China), combines a RGB-D camera (Intel, Santa Clara, CA, USA) with bone-conducting headphones (AfterShokz, East Syracuse, NY, USA), which we use to provide feedback. The computing component is a lightweight laptop (Lenovo, Beijing, China) carried in a backpack. These glasses are connected to a laptop using a single USB3 connection. The reduced amount of components and cabling makes the system ergonomic and easy to use.

Our key perception component, the RGB-D camera, is an Intel RealSense device model LR200 [44]. RGB-D refers to a red, green, blue and depth camera and defines a camera that provides both color estimates as well as distance estimates in each pixel, usually aided by some sort of active infrared illumination source.

The depth camera technology used in the LR200 employs a hybrid design that combines classical stereo triangulation with the projected pattern technique. The LR200 uses two infrared cameras that are used to triangulate the depth perception, in a classical stereo setup. The camera includes hardware to solve the correspondence problem and directly delivers a per-pixel distance estimation in millimeters.

The LR200 also incorporates a laser projector that illuminates the scene with a pseudo-random pattern of dots, in a way analogous to the original Kinect cameras. For pure projected pattern-based cameras, the pattern is required to solve the correspondence problem and triangulate the distance. In the LR200, however, the projected pattern only has an assisting role, increasing the amount of textures on the image. This means that the LR200 is able to provide good depth estimates at distances and illumination conditions under which the projected pattern would not be visible—i.e., outdoors—albeit at a reduced precision. Furthermore, the LR200 suffers from no interference if more than one camera is observing the scene.

Regarding the drawbacks of the LR200, the RGB camera within the LR200 has a diminutive lens aperture, which provides poor image quality in low light situations. We have also observed that the dots projected by the camera can be seen as specular freckles on the RGB camera, specially in low light conditions.

We provide feedback to the user by means of bone-conducting headphones integrated within the glasses. Those transmit the sound to the inner ear through the skull, with the transceiver placed on the zygomatic bone (also known as the cheek bone). While sound quality is generally deemed to be lower than standard headphones, bone-conducting headphones do not obstruct the ears, allowing the users to hear the environment around them.

Our software does not have high performance requirements, and also it needs no specific hardware other than a Nvidia GPU to process the deep learning model. For our user tests, we used a 1.9 kg notebook equipped with a Core i7 5500U CPU and a GT840M GPU. This system includes processing power and battery in one unit and allows for a compact, ergonomic and robust solution for experimentation. However, we expect this system to be deployed using specific embedded hardware; to this end, we have also tested the system and evaluated its performance using an Nvidia Xavier [45], which is a compact system powered by an ARM CPU and a powerful Nvidia GPU.

### 3.2. Software Components

Our prototype uses Ubuntu 20.20 Focal Fossa as our operating system to house our software components and the Robotic Operating System (ROS) [6] to connect them.

**Figure 4:**
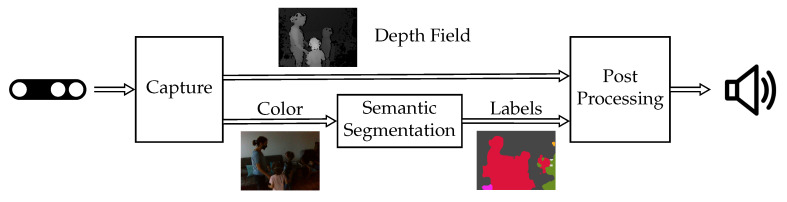
Software architecture. The capture module is implemented in C++ and provides aligned depth and color images. The color image is used by the semantic segmentation module, coded in PyTorch, to generate pixel-wise object class labels. The post-processing module combines the depth field and the label information to provide alerts only for close persons. Communication is handled by the Robotic Operating System.

These can be divided into three main components, as seen in Figure 4. The module that captures data from the RGB-D is implemented in C++, using the librealsense library [46]. The key perception algorithm is based on deep learning and is implemented in Python using PyTorch [47]. The camera interface, post-processing and audio output feedback are implemented in C++ and use OpenCV [48] and OpenAL [49]. To communicate with those components, we use the Robotic Operating System (ROS) [6].

The Robotic Operating System (ROS) is a communication framework used to connect several software components using a message-passing architecture. ROS is ideal for our use case, as it provides native messages types to communicate both RGB images as well as depth fields. Furthermore, ROS messages provide a translation functionality between C++ data structures obtained from librealsense to the Python data structures required by the PyTorch deep learning module. In addition, ROS handles buffering and synchronization problems, allowing the system to run as quickly as possible in real time. By using ROS, we avoided the need to translate the original PyTorch model in Python to a C++ equivalent.

The data capture module uses the librealsense library to access the LR200 camera and capture two of its provided streams: the color stream and the depth_aligned_to_color stream. The color stream provides 640 × 480 pixels of RGB data at 30 frames per second, while the depth_aligned_to_color stream provides per-pixel depth estimates, in millimeters, as a 640 × 480 field of 16 bit values, also at 30 frames per second. In this case, the depth field is already aligned in a per-pixel basis to the color image, so no extra translation is needed. The data capture module labels timestamps for both the captured RGB and depth images and sends the RGB image to the semantic segmentation module.

**Figure 5:**
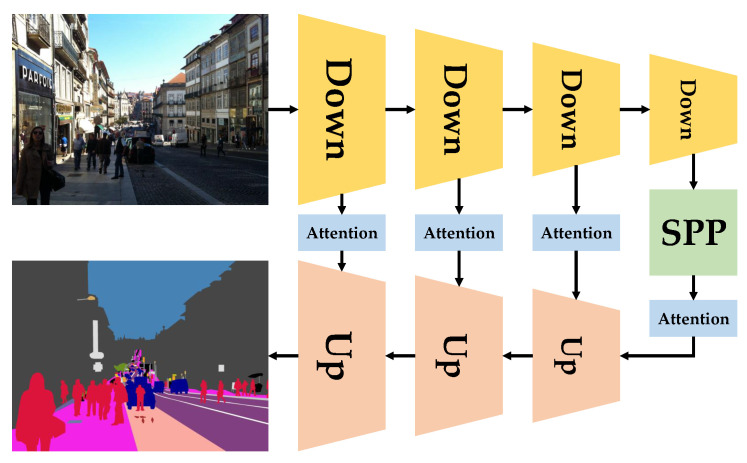
The real-time SwaftNet [2] architecture for swift and accurate semantic segmentation. The downsampling and upsampling paths are connected with attention operations to improve the detail-sensitivity.

We use the real-time SwaftNet model, which was previously developed in the DS-PASS system [2], to sense the surroundings; this model is capable of predicting high-resolution semantic segmentation maps both swiftly and accurately. As it is shown in Figure 5, the SwaftNet architecture is built on an efficient U-shaped structure with channel-wise attention connections based on squeeze and excite operations [50]. In this way, the attention-augmented lateral connections help to spotlight spatially-rich features from the downsampling path, which enhances the detail-sensitivity of semantic segmentation, which is critical for social-distancing detection. Besides this, the spatial pyramid pooling (SPP) module acts as an instrument to enlarge the receptive field before passing features through the shallow lightweight upsampling path for the final pixel-wise classification [2].

SwaftNet is trained on Mapillary Vistas [3], which is a street scene dataset that includes many images captured by pedestrians on sidewalks. In addition, we use a heterogeneous set of data augmentation techniques that are of critical relevance to the generalization capacity in unseen domains [51]. Thereby, the semantic segmentation module performs robustly with glasses for blind people.

The post-processing module receives a timestamped field with labels from the semantic segmentation module and retrieves the depth field with the corresponding timestamp from the data capture module.

Each processed image will create a single beeping signal. Based on prior work, we fix this signal shape to a pure sinusoidal tone of 20 ms in length. We found that this length is sufficient to be perceived but short enough not to mask ambient noises.

As we emit one beep for each processed image, the frequency of the beeping depends ultimately on the processing power of the computing device and is limited to a maximum of 10 beeps per second.

The three parameters we use to modulate the beeping signal are its frequency, its volume and its spatial location. To obtain the corresponding values for those parameters, we apply a light post-processing step. We discard pixels that are not classified as persons, pixels whose distance is not provided by the depth camera, pixels closer than a minimum distance ($D_{min}$) set to 50 cm and pixels further away than a maximum distance ($D_{max}$) set to 150 cm. Of the remaining pixels, we only retain the 25% that are closest to the camera; thus, we focus on the closest person visible. The system is not very sensitive to this threshold, and any value between 10% and 50% performs well for the purpose of focusing on the closest person.

The volume is proportional to the number of pixels retained and reaches a maximum level when 5% of the image pixels are still retained. The stereoscopic sound allows us to signal the sound as if it were coming from a specific direction. The direction of the sonification is calculated by averaging the horizontal image coordinate of all remaining pixels.

Finally, the frequency of the tone is mapped to indicate urgency. Our system aims to be unobtrusive during most daily activities but to be intrusive—even to the point of being annoying—if it finds a person in front of the user that is too close to them. High frequencies are known to be more annoying than low frequencies; thus, we increase the frequency when we consistently detect a person in front of the user for longer periods of time, thus forcing the user to take action and increase their physical distance. By starting the beeping at a lower frequency, we prevent spurious false detections from being overly inconvenient.

The frequency mapping works as follows. Each selected pixel whose location was not selected in the previous frame starts with a frequency of 220 Hz; this frequency increases exponentially at a rate that is doubled each second. The frequency reaches a maximum at 1760 Hz, which is reached 7 s after finding a person within the warning range. The final notification tone simply averages the frequency of all selected pixels.

**Figure 6:**
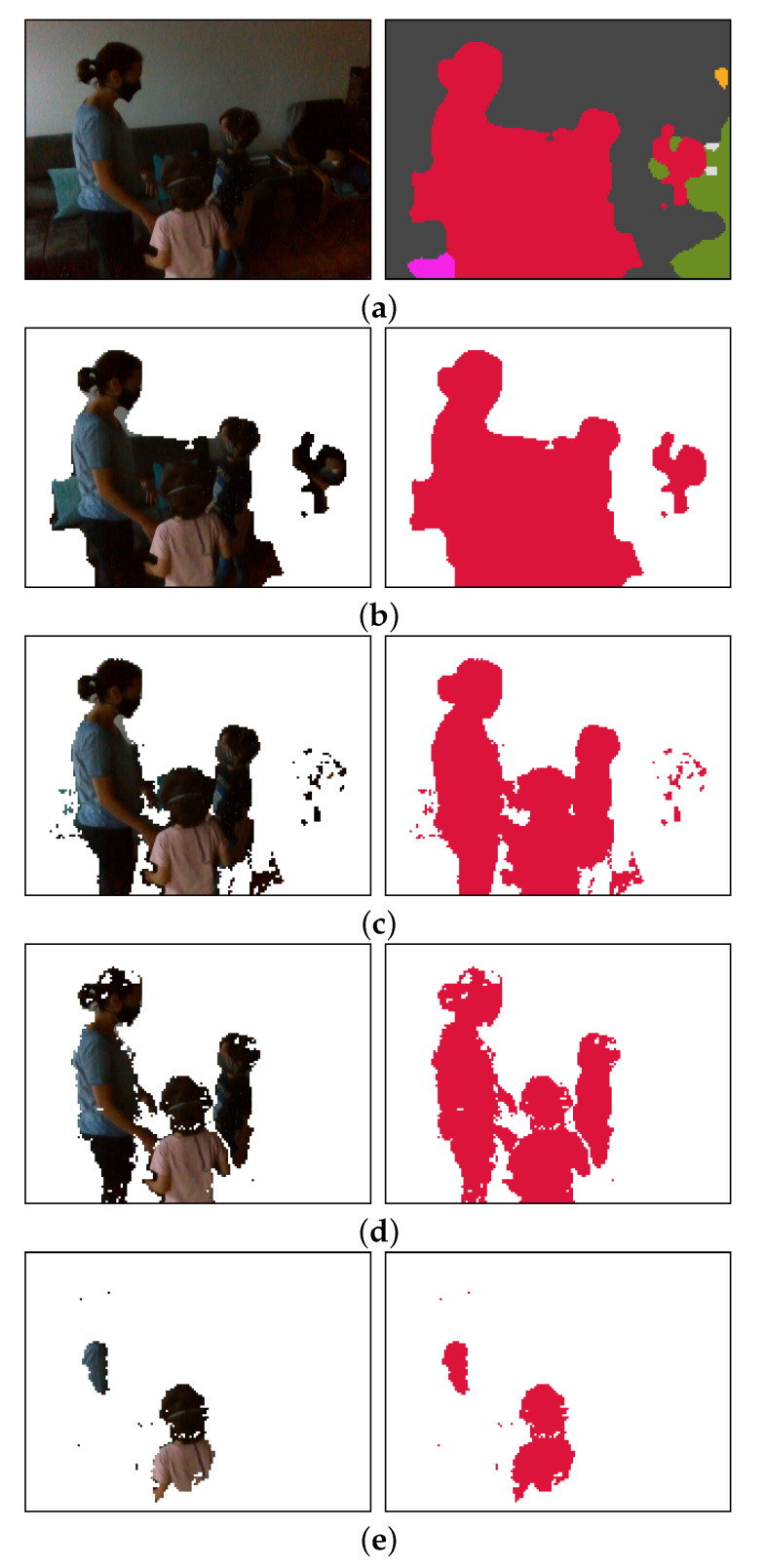
Image post-processing steps. (**a**) Source image from the color camera (**left**) and its semantic segmentation (**right**); (**b**) pixels not labeled as persons are filtered out; (**c**) pixels beyond 1.5 m are filtered out; (**d**) pixels closer than 5 m are filtered out. This includes pixels whose depth value cannot be calculated (reported distance 0 m); (**e**) Of the remaining pixels, only the 25% closest to the camera are retained, which are the pixels that contribute to the sonification.

An example of the post-processing process can be seen in Figure 6.

## 4. Technical Evaluation

The semantic segmentation model has the task of finding persons within the 2D image. We used SwaftNet as our semantic segmentation model and trained it on Mapillary Vistas [3], which is a dataset designed to recognize objects on a street-level context and includes the person class, which we used for our system. Table 1 displays the class-wise accuracy and the mean Intersection over Union (mIoU) results fir the Mapillary Vistas validation dataset. As can be seen from Table 1, SwaftNet achieves good segmentation accuracy on the most navigation-critical classes such as traffic light (62.8%), sidewalk (68.8%), person (69.9%), rider (47.3%) and crosswalk (62.3%).

**Figure 7:**
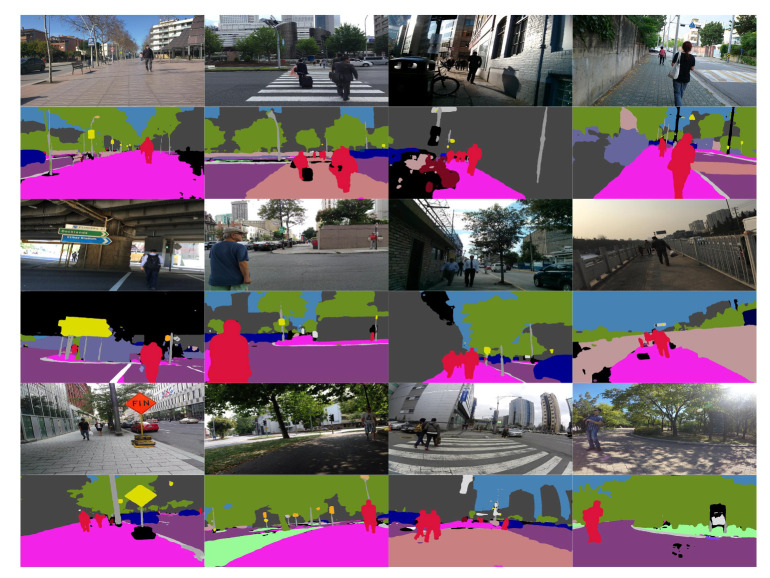
Qualitative examples of semantic segmentation with SwaftNet [2] on images taken from the perspective of pedestrians walking on sidewalks and crosswalks.

Furthermore, in Figure 7, we display a set of qualitative segmentation examples on images taken from the perspective of pedestrians walking on sidewalks and crosswalks. These images were extracted from the test subset of Mapillary Vistas, which represent unseen scenes to the SwaftNet model. It can be seen that SwaftNet achieves robust semantic segmentation, even in low and complex illumination conditions. Especially, it allows the accurate detection of surrounding persons at the pixel level, which largely facilitates our social distancing detection.

Mapillary Vistas contains views from multiple cities around the world, but those images belong to the very specific street view scenario and lack challenging images from scenarios in which we aim to apply our method; e.g., egomotion indoors and outdoors. To assess the real-world semantic segmentation accuracy of our trained SwaftNet and determine its performance in scenarios in which it was not trained, we evaluated SwaftNet on the PASS dataset [51], which was captured by a wearable navigation assistance system. The PASS dataset better reflects the targeted scenarios, as it was captured using head-mounted lenses; thus, it was an ideal dataset to estimate the real-world semantic segmentation of our system.

**Figure 8:**
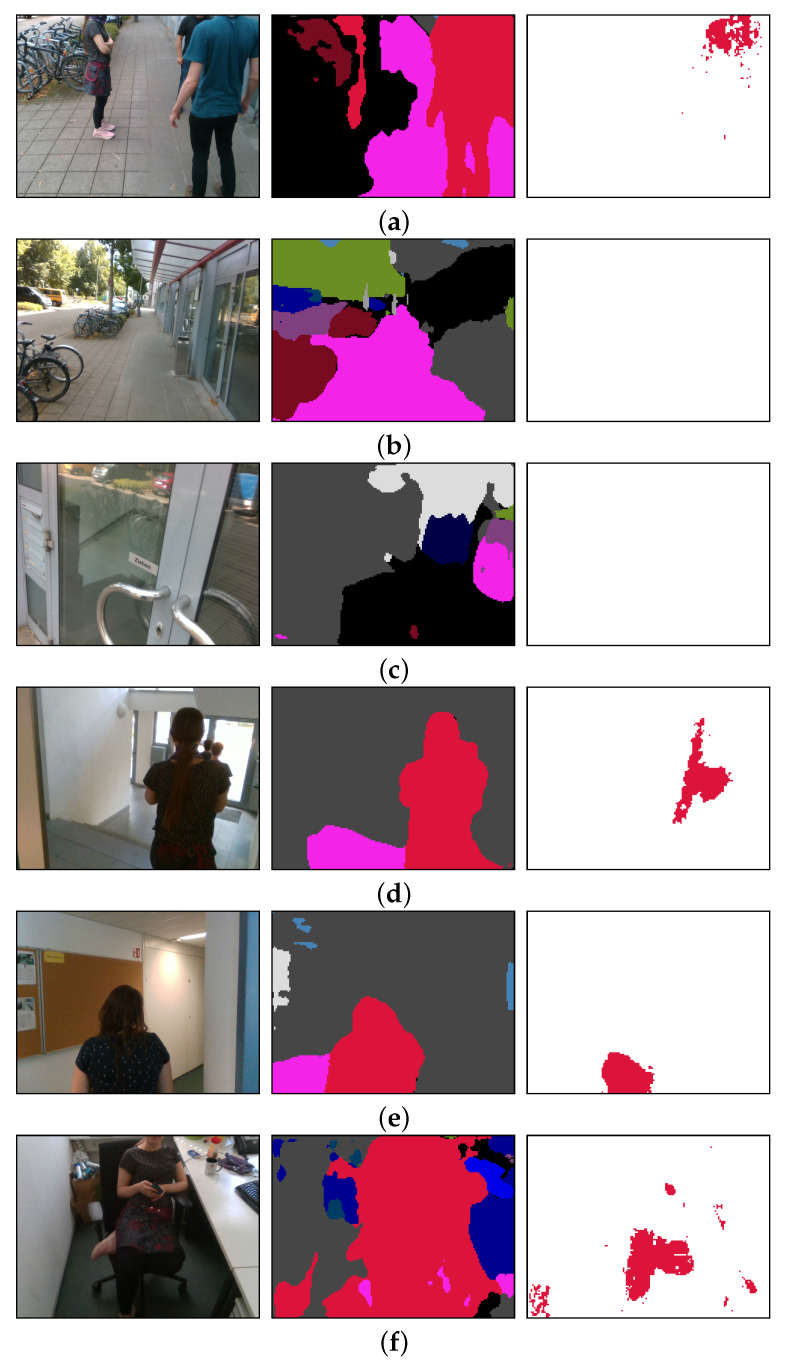
The color image (**left**), the segmentation mask (**middle**) and the sonified pixels (**right**). Persons are labeled in red. Our system works outdoors (**a**–**c**) and indoors (**d**–**f**). Sonification only occurs when persons are present in the image. (**a**) A group is talking in front of a door; (**b**) the same scenario without people, which produces no sonification; (**c**) despite being close to a door, there is no sonification; (**d**) following a person; (**e**) following a person; (**f**) talking to a person.

In Table 2, we evaluate the trade-off between accuracy and latency when running our model on our portable platform. We leveraged the fully convolutional architecture of SwaftNet, which allowed us to use different input image resolutions without the need to retrain the architecture. At higher resolutions, the segmentation was more accurate, while at lower resolutions, the model ran faster. In our field tests, we used an input resolution of 320 × 240. This provided sufficient accuracy when recognizing persons closer than 1.5 m, as seen in Figure 8, and the delay allowed for almost 10 frames per second, which provided sufficiently fast feedback to the user. When tested on the Nvidia Xavier platform, our algorithms ran significantly faster than on the test laptop, showing that the embedded platform is not only more portable but also exhibits better performance. On Xavier, SwaftNet maintains more stable inference speeds and is able to render semantic perception at higher resolutions in near real time. It should be noted that, although the SwaftNet is trained with street-view images, it generalizes well to diverse unseen domains, even for indoor scenes in which persons can be robustly segmented and filtered out for sonification.

## 5. User Study

We conducted a qualitative study with five users, as recommended by Jakob Nielsen [52]. This also included blindfolded users and at least one blind person [53]. Our purpose was to obtain insights regarding the acceptance of our idea, the prototype and its future development. Due to the current imposed restrictions due to COVID-19, our user study was limited to one blind person (P1B), with the other four users being sighted (P2-P5). There were four males and one female, with an average age ranging between 30 and 40 years.

We conducted tests in two different environments: indoors in an office and outdoors on a university campus. In all cases, one person tested the device and three to four people simulated different social scenarios, including but not limited to the following:

- Person obstructing a door, while the blind user aimed to enter the door;
- Group of people having a conversation in the middle of a walkway which the blind person wanted to walk across;
- Persons waiting in a queue at the entrance of a door;
- Having a group conversation, including the blind user;
- Navigating through the building and across the street.

We first asked the users to sign a data protection statement and then allowed a few minutes for the users to familiarize themselves with the interface of our prototype before starting the tests. Immediately after the test, we applied the RTLX questionnaire to measure the cognitive load, the System Usability Scale (SUS) [54] to measure the usability and a self-created questionnaire with five system-related questions.

The hardware was disinfected thoroughly between tests. All participants wore masks throughout the duration of the study. Due to the insufficient ventilation inside the backpack used in the study, combined with air temperatures of up to 32 ${}^{{}^{\circ}}$C outdoors, the laptop often reached throttling temperatures. This, combined with a software bug, created an excessive delay between the processing of an image and the audio feedback, reaching up to 500 ms. Once the bug was resolved and sufficient ventilation was provided, the delay between the image and audio returned to an expected value of about 100 ms.

### 5.1. Motivation

We recorded the conversations we had during the test as part of our test protocol. Our blind user was so enthusiastic about the social distancing problem and prototype itself that we consider it valuable to cite some of his spontaneous comments verbatim. These comments show the value of our system for both people with blindness and sighted people:

*“This [social distancing] is the main problem, because physical distancing became social distancing. In general, I hate social distancing because […] we don’t want to distance socially. It’s physical distancing, but now it is the case that physical distancing became social distancing. People don’t speak, I cannot hear them, I cannot keep my distance and it’s difficult in trains, in the tramway, in shops. Sometimes people help, but in general they don’t communicate because they wear a mask, so they don’t communicate, I can‘t hear them, it’s like Ghostbusters a little bit.”*

*“[After a long test session] It only recognizes persons. Fascinating. I think it‘s a very good start. And I think that if you implement it as an App, I would want to pay for that. Because this […] distancing is so difficult for us. I hate it from morning to night.”*

*“I believe that such an aid would be very very helpful to keep distance. Not only because of Corona, but distance, if you are warned […] then I know that I have to be careful with my cane.”*

Two other participants (P2,P3) said that the system would also be “good for sighted people to be able to judge distances better” (P3). P2 reported an incident in the supermarket in which a cashier was unfriendly to her because she accidentally came too close to another customer.

### 5.2. Cognitive Load

**Figure 9:**
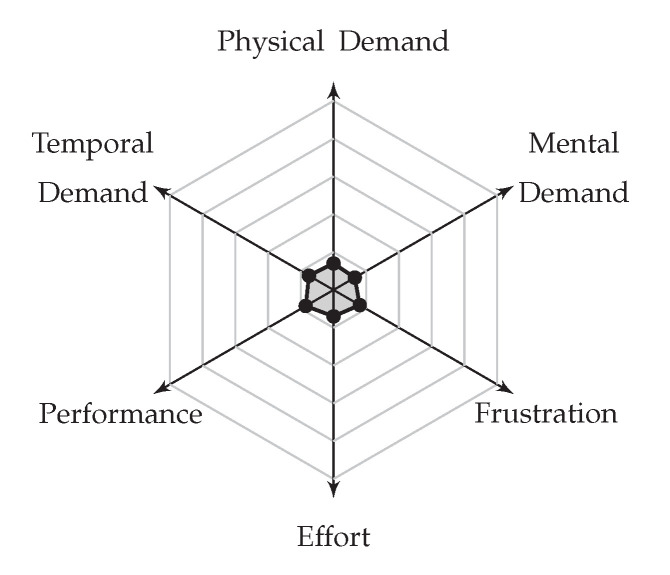
The Raw NASA Task Load Index (RTLX) measures the workload needed to operate the system. Our prototype requires a low averaged raw workload index of 3 (out of 20).

In the RTLX test that we administered, users were asked only to provide ratings, without weights. Averaging the ratings over the six factors (see Figure 9) and for all participants, we obtained an average of 3 out of a maximum of 20, with a standard deviation (SD) of 1; this represented the equivalent of a NASA-TLX score of 15 (or 15%). According to Grier [7], who included RTLX scores in her evaluation of cumulative frequency distributions, a workload of 15 represents between 25% and 50% of all cases for the 33 daily activity tasks analyzed (where the minimum workload was 7.20 and the maximum was 37.70). We thus argue that our system does not overly increase the cognitive load but adds just enough for the user to remain alert and motivated.

### 5.3. Usability

We measured the usability of the system using the System Usability Scale (SUS), giving a general assessment of the perceived usability of the system.

The SUS score obtained by our five participants ranged between 77.5% to 95.0%, with a mean of 86.5 (SD 7.2). The blind participant gave the highest SUS score. According to Bangor et al. [55], who analyzed 2324 surveys from 206 studies, “the best quarter of studies range from 78.51 to 93.93”. This places our system quite high in terms of perceived usability. We must say, however, that the prototype was turned on and off—and thus controlled—by the experimenter, not by the users. This could have had a positive impact on the perceived usability.

### 5.4. User Comments

We analyzed the comments made spontaneously during the study and the answers to the free text question “What did you/didn’t you like about the system?” We report the results below.

In terms of the positive aspects, the idea of helping blind people to achieve physical distancing was viewed very positively, and the prototype was deemed to cover all the functionality required for this application. The fact that it reacts to people and nothing else was appreciated very much by the blind participant. The glasses, albeit still a little bit bulky, were deemed to have a very nice design and to be sufficiently light. The inclusion of the bone-conducting headphones was also very appreciated. The interface, which warns only when the distance is below a certain threshold, was received positively.

However, there were complaints about the need to wear a backpack. There was a general consensus that this application must be integrated into a smartphone for people to use it. The frequency mapping was understood but deemed not to be intuitive. The processing platform also became excessively hot; furthermore, the audio volume was not automatically adjusted and was too loud indoors.

In answer to the question “Did you find the system useful?”, on a scale from 1 (very useful) to 5 (very unhelpful), the mean answer was 1.4, with a standard deviation of ±0.54. Thus, the participants found the prototype useful.

In answer to the question “What did you find better: walking using the system or without?”, All participants answered that they preferred to walk using the system than without.

## 6. Conclusions

In this work, we have developed a wearable system to help blind and visually impaired individuals maintain safety-critical social distancing, which is a highly challenging task during the times of COVID-19. We used a head-mounted glasses system with a RGB-D camera that combines stereo matching and pattern projection for dense depth estimation. We leveraged an efficient semantic segmentation algorithm that facilitated the detection of persons both swiftly and accurately, whose 3D positions can be measured by using pixel-wise segmentation maps with aligned depth information. We provided acoustic feedback when the detected persons were in close proximity through the bone-conducting headphones on the glasses. A variety of experiments and one user study demonstrated that the system was reliable and easy to use, with a low cognitive load. The comments of the blind user highlighted the value of our system throughout the pandemic and beyond.

Our current prototype used a laptop as a processing platform to validate the concept, but for it to be practical, this processing platform must be integrated in a smaller package. We took the first steps towards this integration process by evaluating our software on a Nvidia Xavier, showing excellent results. With regards to future work, we aim to evaluate even smaller integration possibilities; e.g., by migrating our code to TensorFlow Lite [56] and testing edge platforms such as Coral [57] and Movidius [58].

## Figures and Tables

**Table 1:** Semantic segmentation accuracy for the Mapillary Vistas dataset [3].

**Pole**	**Street Light**	**Billboard**	**Traffic Light**	**Car**	**Truck**	**Bicycle**	**Motorcycle**	**Bus**	**Sign Front**	**Sign Back**	**Road**	**Sidewalk**	**Curb Cut**
47.5%	35.8%	43.4%	62.8%	90.3%	70.4%	55.9%	59.1%	75.1%	69.5%	38.7%	88.6%	68.8%	14.7%
**Plain**	**Bike Lane**	**Curb**	**Fence**	**Wall**	**Building**	**Person**	**Rider**	**Sky**	**Vegetation**	**Terrain**	**Marking**	**Crosswalk**	**mIoU**
17.4%	37.3%	55.5%	55.0%	46.7%	86.6%	69.9%	47.3%	98.2%	89.7%	63.7%	53.5%	62.3%	59.4%

**Table 2:** Real-world segmentation accuracy (in mean intersection over union (IoU)) on the PASS dataset [51] and delay analysis (in milliseconds per frame).

Resolution	Mean IoU	IoU of Person Seg.	Delay on the Laptop	Delay on Nvidia Xavier
960 × 720	68.3%	81.8%	600.6 (±6.7)	108.9 (±0.36)
640 × 480	66.9%	80.4%	292.1 (±5.7)	57.9 (±0.94)
480 × 360	55.1%	77.7%	184.0 (±8.1)	52.6 (±1.2)
320 × 240	50.8%	63.3%	107.9 (±4.5)	46.7 (±1.0)
